# Supplementary material for: Measurement invariance tests of revisions to archaically worded items in the Mach IV scale
Source: PLoS One. 2019 Oct 16;14(10):e0223504. doi: 10.1371/journal.pone.0223504 (PMC6795441; doi:10.1371/journal.pone.0223504)
Supplement: Attachment — Submitted filename: Review MACH.docx [file pone.0223504.s001.docx]

**General comment:**

The authors conduct exploratory and measurement invariance analyses of a scale which is intended to measure the construct Machiavellianism. More specifically, they examine the impact of a rewording of some of the items on the structure and measurement properties of the scale.

The method of analysis and the rationale underlying it are described clearly. However, I think that some of the conclusions which are drawn from the analysis are debatable. For instance, an initial step of testing weak measurement invariance, results in a mediocre model fit. Given this (mis)fit, the subsequently drawn conclusions from the model comparisons do not necessarily seem stringent (see comments below). Moreover, it should be stated that the reported fit-indices are likely to be biased upward, given that preliminary model selection was already applied (that is, a preliminary elimination of “bad” items was conducted). Finally, I also think that the usage of a 4-6-factor solution for a test comprised of less than 20 items is problematic (see comments below) and should be discussed appropriately.

**Specific comments**:

Introduction: The paper would benefit from providing some brief review of the theory underlying the construct of Machiavellianism. In addition, providing the content/wording of all items would be helpful in understanding the proposed factor structure.

34: Factor indeterminacy is a general problem prominent in any FA model.

35-38: This suggests large variation between different (study) populations.

51:53: This could be tested via factorial invariance. Are there any corresponding results in the literature. Why does the current study not include such a test of invariance across gender?

The aim seems to be to optimize the MACH for student populations, as a lot of the presented arguments are tied to changes in student populations, i.e. a change in the proportion of female and international students. This should be mentioned as a limitation because I don't think that students are the primary focus for the use of a diagnostic instrument for Machiavellianism.

115: I would avoid the usage of cut-off scores like .3. On the one hand, they are somewhat arbitrary. But more importantly: It can be shown that even small loadings (below .3) can have a large impact on the inference of the factor scores when diagnosing test takers (see e.g. Jordan & Spiess, 2019).

133: It is correctly stated that alpha only provides a lower bound for the overall reliability. However, even if alpha were sufficiently high, it would - in my opinion – still be of limited use because its reference point is a formally defined "overall" true score which in this case is a mixture of multiple (6) dimensions.

141: There are more reliable approaches to the determination of the number of factors like e.g. Horn's parallel analysis (PA) or the more recently developed “deterministic counterpart” based on random matrix theory (see Dobriban & Owen, 2019). The authors already mentioned large variations of the number of extracted factors across different studies. Hence, I wonder if some part of the variation could be explained by "suboptimal" extraction criteria. In any case, I would suggest the use of PA to determine the number of factors.

162: The authors should expand their argument on negative loadings. In general, negative loadings are not a problem unless they contradict the factor label (i.e. an item in an intelligence test with negative loadings would contradict the label "IQ" as test takers with lower IQ would score higher on the item).

233: The examination of normality is not necessary. We already know that normality can't hold due to the discrete (1 to 7) format of the responses.

246-250: How was the CFA specified? By usage of the exact loadings from the EFA or by treating loadings below .3 as zero? In addition, heywood cases should not arise under ML-estimation. If the ML method did not converge (to which I think the heywood case refers), then this points to problems in the specification of the model.

263: I do not understand what is meant by "the covariances of ... were unidentified". Does this mean that the estimated factor covariance matrix was not positive definite?

264: It seems problematic to use such a few number of items per factor. Measuring a factor by 2-5 items is tantamount to produce scorings with low reliability.

275: "unidentified": It would be helpful to distinguish an unindentifiable case from a case, wherein the numerical optimization did not converge and/or provided inadequate estimates (e.g. negative variances). Which type are the authors referring to?

308: The problem with the approach for determining various sorts of measurement invariance is that the baseline model does not provide a good fit. Hence, although it seems that given the baseline model, further restrictions are possible without substantially lowering the CFI, the fit of the baseline model itself is somewhat questionable. Given these doubts, I think that the subsequent comparison of error variances is problematic as it relies on a good overall model fit (and not just on a good fit relative to a model with mediocre fit).

372: This line of reasoning should be strengthened by computing model based reliability estimates. In addition, to demonstrate an effect of item wording, I would recommend to append the paper with a test of the treatment effect. That is, are there any difference in the distribution of the responses to an item between treatment and control group?

395: In the general discussion, critique of the MACH is mentioned. I think it would be important to add and discuss the following topics:

- How can we account for (or interpret) the large variations (e.g. number of factors; number of retained items) across studies?

- If the wording of items has an impact on the reliability (or potentially even on the factor structure) of the scale, then this leaves "us" with measurement devices (self-report questionnaires) which are very fragile. Hence, I think a point could be made here in favor of moving towards other (perhaps more costly) measurement devices (e.g. actual observation of behavior; using multiple sources of information etc.).

- Given the relatively large number of underlying factors (4-6), the researcher/practitioner has the option to

a) either compute an inhomogeneous overall score which refers to a (difficult to interpret) mixture of (4-6) constructs

or

b) to compute dimension specific scores (4-6).

However, choosing b) basically boils down to measure a latent construct by only a few items. Hence, the scores are highly unreliable.

- Related to the previous point, but broadening the scope: A topic of central importance in the analysis of the replication crisis in psychology referred to the role of the measurement error (see e.g. Loken & Gelman, 2013), i.e. measurements in psychology are in general rather noisy. I think that by using such short (on average 4-5 items per factor) subscales, classical test theory would predict unreliable, noisy measurements. Thus, their subsequent usage might entail all of the problems which were discussed within the context of the low replicability of psychological science. Hence, I regard this as an additional argument against the usage of the scale.

References:

Dobriban, E. & Owen, A. B. (2019). Deterministic parallel analysis: an improved method for selecting factors and principal components. J. R. Stat. Soc. B, 81: 163-183. doi:[10.1111/rssb.12301](https://doi.org/10.1111/rssb.12301).

Jordan, P. & Spiess, M. (2019). Rethinking the interpretation of item discrimination and factor loadings, Educational and Psychological Measurement.

Loken, E., & Gelman, A. (2017). Measurement error and the replication crisis. Science, 355(6325), 584–585.
