# Supplementary material for: Measurement invariance tests of revisions to archaically worded items in the Mach IV scale
Source: PLoS One. 2019 Oct 16;14(10):e0223504. doi: 10.1371/journal.pone.0223504 (PMC6795441; doi:10.1371/journal.pone.0223504)
Supplement: Attachment — Submitted filename: Response to Reviewers.docx [file pone.0223504.s003.docx]

**Response to Reviewers**

Reviewer #1: The authors have addressed most of the points which were raised. They also provided convincing arguments for their approach. Some comments/suggestions remain:

General:
I can relate to the authors aim of providing a polite and respectful critique of the MACH. However, in my opinion, in some cases this aim has led to formulations within the paper which do almost give an ambiguous notion about the usefulness of the scale, when in fact the usefulness of the scale could have heavily been called into question. I realize that a direct statement of this critique risks being interpreted as disrespectful – however, I think that the benefit, that readers/researchers get a clear-cut impression on the properties of the scale outweighs this risk. However, this is just a matter of opinion and there is no need for the authors to address this point (in fact, the revised version contained clearer formulations of the central properties of the scale.)

Authors’ response: We thank the reviewer for understanding our plight. We “walked a tightrope” a bit but are glad that the reviewer is pleased with our revisions that do indicate our point of view of the scale.

Specific:
l 190+: Maybe I do not understand this correctly, but the argument in terms of the negative loading does not seem convincing to me. If it is just a problem with respect to the computation of the sumscore, then one could resolve this issue by reversing the item (or by scoring it with a negative weight). A more convincing argument would deduce a contradiction between the quantity the factor is supposed to measure and the anticipated sign of the item that loads on this factor.

Authors’ response: Ah…now we understand. We regret not being clearer in the original version of our paper when we simply listed "reverse coded" next to some example items. We actually recoded items that were reverse scored before submitting them to EFA and the CFA. We have added the following to lines 148-149: "All items were corrected for reverse scoring before the EFA was conducted.” Additionally, on lines 205-206 we added the following: “All items were corrected for reverse scoring before the CFA was conducted.” So, after reverse coding the reverse scored items we found that some of the items load negatively on some factors that also had positively loading items. This is not good in that items designed to measure facets of a construct should be positively correlated with that facet as well as with other items measuring the construct. Some of the items in the Mach IV are not positively correlated with each other. We thank the reviewer for pointing out how we may have just skipped by this without a proper explanation and we hope that our corrections to the paper and our explanation make things a bit clearer now.

l 299+: It might be confusing to mention a sixth factor “However, the covariance between Factor 4 and Factor 6 was unidentified” within a five-factor model. Perhaps the authors could add a short statement to clarify this. (I guess, it refers to the elimination of the former fifth factor which was related to a Heywood case)

Authors' response: This is a very good point. We have added the following to lines 277-278 in the hopes of clarifying our nomenclature: "In the following CFA we refer to the items of the Mach IV as well as the factors that emerged from the EFA by our original numbering system."

l 417+: I appreciate the added information on the treatment-control comparison. However, in order to avoid filtering the results in terms of statistical significance, I would recommend to provide a table which contains the results of the comparisons on all relevant items. That is, the table should also include the results of nonsignificant comparisons. Ideally, an estimate of an effect size measure (computed for significant as well as for nonsignificant comparisons) should also be added to enhance the evaluation of the treatment.

Authors' response: We have added the table that is suggested. In addition to the information requrested we have added a column in the table to indicate if an item was discarded or retained from the measurement invariance tests and a column indicating if the item was a revised item or an original item. To match this new Table 6 we have rewritten the verbiage on lines 415-425 as follows: "Additionally, in an item-by-item comparison of scores in the control and treatment groups, three items had unequal variances by virtue of Levene’s test. All were revised items. Item 6 about vicious streaks (*F* = 4.234, *p* < .05), item 11 about Barnum and suckers (*F* = 4.221, *p* < .05), and item 18 about men working hard (*F* = 18.130, *p* < .001) each had a larger spread of scores in the revised version than in the original version of the items. Item variances for other items in the control and treatment groups were not different. There were also significant differences in the means for those three items. Item 6 (*t* = -4.234, *df* = 472.66, *p* < .001, Cohen's *d* = .381) and item 18 (*t* = -6.425, *df* = 466.15, *p* < .001, *d* = .580) had higher means in the revised versions of the items and item 11 (*t* = 7.992, *df* = 476.49, *p* < .001, *d* = .727) had a higher mean in the original item. Item means for other items were not different. See Table 6 for the results for all items." Our new table begins on line 426. Thank you very much for your insightful suggestions and keen eye.
